# Camera-Based Dynamic Vibration Analysis Using Transformer-Based Model CoTracker and Dynamic Mode Decomposition

**DOI:** 10.3390/s24113541

**Published:** 2024-05-30

**Authors:** Liangliang Cheng, Justin de Groot, Kun Xie, Yanxin Si, Xiaodong Han

**Affiliations:** Dynamics and Vibration Group, Engineering and Technology Institute Groningen, Faculty of Science and Engineering, University of Groningen, 9712 CP Groningen, The Netherlands; j.de.groot.21@student.rug.nl (J.d.G.); k.xie@rug.nl (K.X.); y.si@rug.nl (Y.S.); clint.han@rug.nl (X.H.)

**Keywords:** vibration, modal analysis, camera-based measurement, deep learning, CoTracker, dynamic mode decomposition

## Abstract

Accelerometers are commonly used to measure vibrations for condition monitoring in mechanical and civil structures; however, their high cost and point-based measurement approach present practical limitations. With rapid advancements in computer vision and deep learning, research into tracking the motion of individual pixels with vision cameras has increased. The recently developed CoTracker, a transformer-based model, has demonstrated excellence in motion tracking, yet its performance in measuring structural vibrations has not been fully explored. This paper investigates the efficacy of the CoTracker model in extracting full-field structural vibrations using cameras. It is initially applied to capture the dense point movements in video sequences of a cantilever beam recorded using a high-speed camera. Subsequently, modal analysis using delay-embedding dynamic mode decomposition (DMD) is conducted to extract modal parameters including natural frequencies, damping ratios, and mode shapes. The results, benchmarked against those from a reference accelerometer and the Finite Element Method (FEM) result, demonstrate CoTracker’s high potential for general applicability in structural vibration measurements.

## 1. Introduction

Dynamic vibration information, such as displacement, velocity, and acceleration, is essential to assessing structural integrity and predicting future conditions within Structural Health Monitoring (SHM) and Predictive Maintenance (PM) practices. Structural responses to external loading can yield critical insights into underlying structural dynamics (e.g., natural frequency, damping ratio, and mode shape) through conducting Experimental/Operational Modal Analysis (EMA/OMA) that reflects the structure’s physical properties such as stiffness [1,2]. For instance, a decline in natural frequency may signify a reduction in structural stiffness, and irregularities in mode shape may signal localized defects. Despite its importance, accurately measuring sufficient dynamic responses of real engineering structures remains a challenge due to limitations in current practical methods.

Contact-type accelerometers are widely considered for vibration measurements in various mechanical, civil, and aerospace applications [3,4]. However, their inherent limitation of single-point, contact-type measurement often renders them incapable of capturing sufficient dynamic information about the structures [5]. For instance, fully understanding the mode shapes of bridges cannot be realized by only placing a few accelerometers. Moreover, the locations of the sensors should avoid the so-called mode nodes to ensure effective data collection. This leads to huge burdens (e.g., high labor/money/time cost) for conducting experimental campaigns of vibration tests using accelerometers. With the rapid advances in high-speed cameras regarding the hardware, camera-based motion capture techniques have gained considerable attention from researchers and managers in structural vibration monitoring owing to their simple, cost-effective, and non-contact sensing method [6,7,8].

The efficiency of vision-based approaches, which take advantage of remote sensing, full-field measurements, and high precision, have been identified in many applications [9,10,11,12,13,14,15,16,17]. A variety of vision-based techniques have been designed and applied for displacement monitoring such as digital image correlation (DIC) [9], template matching [10], optical flow algorithms [11], feature tracking [12], up-sampled cross-correlation (UCC) [13], mean-shift tracking methods [14], and phase-based methods [15], among others [16,17]. Among these, DIC stands out for its ability to correlate and track image segments over time, while template matching utilizes known patterns to locate corresponding areas in different images. Optical flow algorithms estimate the motion of objects between frames and feature tracking methods follow distinct points or objects within the image sequence. The aforementioned techniques, despite their efficacy in motion estimation within image sequences, present several inherent limitations. Some require the application of painted patterns, which may be impractical for large-scale or in-service infrastructures, while others rely on assumptions of small motion, potentially limiting their performance and applicability in practical scenarios involving complex or large displacements.

A deep learning model called CoTracker [18] for motion tracking, built on a transformer network using specialized attention layers to model the correlation of different points over time, was recently proposed by Nikita, etc. Unlike conventional methods that either employ optical flow for simultaneous motion estimation of all points or track each point separately, which may neglect inter-point relationships, possibly reducing performance, CoTracker addresses this by jointly tracking multiple points throughout a video, which blends ideas from both optical flow and tracking studies. The direct use of pretrained AI model parameters reduces application complexity and enhances the ability to capture structural motion at the pixel level, creating significant potential for a wide range of real-world applications. However, research into the feasibility and performance of CoTracker for vibration measurement in the field of Structural Health Monitoring (SHM) has not yet been explored.

This paper primarily aims to conduct a preliminary feasibility study on the use of the trained CoTracker model for measuring the dynamic vibrations of a cantilever beam in a laboratory setting. The performance of CoTracker is benchmarked against signals measured by a reference accelerometer. Furthermore, the paper discusses the possibility and efficacy of conducting modal analysis to infer the structural dynamic properties (i.e., natural frequency, damping ratio, and mode shape) using a data-driven decomposition approach known as Dynamic Mode Decomposition (DMD) [19,20,21], initially introduced for flow dynamics but now applied beyond this field.

The paper is organized into five main sections. Section 2 provides an overview of the CoTracker model and a detailed description of the methodology is presented in Section 3. Section 4 details a case study on a steel cantilever beam. The results obtained and the comparative benchmarking studies are also presented in Section 4. Finally, conclusions are drawn in Section 5.

## 2. The Co-Tracker Model

The current mainstream motion tracking methods mainly fall into two categories: one is the optical flow method which estimates the instantaneous velocity of all points in the video frame, but it is difficult to estimate long-term motion (especially when there is occlusion and the camera frame rate is low). The other is the tracking method, which involves selecting a limited number of points and directly tracking them over continuous time, but this does not utilize the interaction relationships between different points on the same object. CoTracker, however, employs the concepts of both methods: it uses a transformer to model the correlations of points on the same object and uses a sliding window to track ultra-long video sequences. The input of CoTracker is the video and a variable number of trajectory starting positions, and the output is the entire trajectory of the predefined positions. The main innovation of CoTracker lies in its capacity to utilize both temporal and group attention blocks within the transformer architecture. By alternating between these attention blocks, CoTracker gains a more thorough comprehension of motion dynamics and correlations among points. This approach allows Co-Tracker to tackle the constraints of conventional methods, which concentrate on tracking points separately.

**Figure 1:**
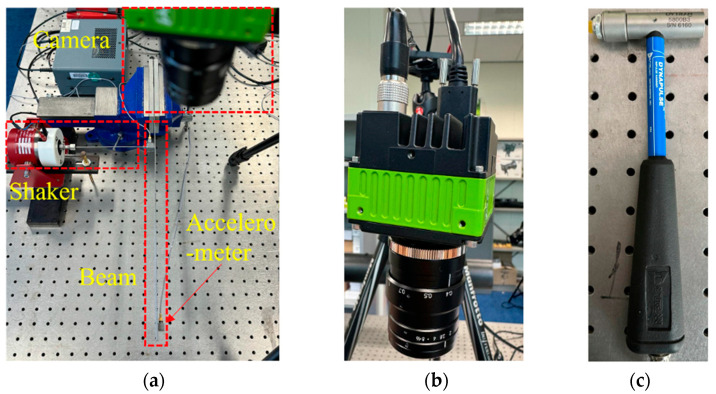
Experimental setup for measuring vibrations on a cantilever beam: (**a**) overall experimental setup; (**b**) high-speed camera with a high-resolution lens; (**c**) impact testing hammer.

Note that the network’s input can be any position and any time within the video sequence. The architecture of CoTracker is presented in Figure 1, and the fundamental principle of CoTracker is detailed in [18] as follows: First, assume that the points are stationary to initialize the point coordinates P, then use a Convolutional Neural Network (CNN) to extract image features Q. A marker ‘v’ is introduced to denote whether the target is occluded. Subsequently, the input token, comprising the point coordinates (P), occlusion marker (v), and image features (Q), is processed by the transformer to model correlations. The output token, consisting of the updated position (P’) and image features (Q’), is then obtained. Furthermore, Co-Tracker offers flexible ways to define the tracking points: grid-based tracking, manual point selection, and user-defined segmentation mask. In this paper, a segmentation mask covering the beam edge is defined.

## 3. Methodology

### 3.1. Experimental Setup

Experiments involve vibration measurement on a cantilever beam under both harmonic excitation due to a modal shaker and free vibration following an impact using a force hammer. The experimental setup is illustrated in Figure 1, where the beam has dimensions of 420 mm in length, 2 mm in width, and 30 mm in height. The sampling frequency of the accelerometer was 8192 Hz, and that of the high-speed CMOS camera with a high-resolution lens for sensor size up to 1” and a focal length of 16 mm was set to 150 Hz.

More details regarding the experimental procedures are summarized as follows: (i) A single harmonic frequency of 20 Hz from the modal shaker will be generated to initially excite the beam, as shown in Figure 1a. Subsequently, the high-speed CMOS camera will be used to capture the sequence of vibration images after conducting camera calibration. (ii) Next, to extract the full-field vibrations from the observed beam structure, a segmentation map consisting of a 500-pixel side edge will be created. The segmented pixels will then be inputted into the trained CoTracker model to extract the time-domain vibration signals from the entire side edge of the beam. This will allow for better visualization of the vibration modes after conducting modal analysis in the next step; the measured vibrations using the camera will be benchmarked with that of the accelerometer (see Figure 1a). (iii) Further validations on the feasibility of the proposed methodology are conducted via the measurement of vibrations under hammer impact and the relevant modal parameters including natural frequency, damping ratio and mode shape will be identified using a data-driven approach, that is, delay-embedding dynamic mode decomposition.

### 3.2. Camera Calibration

**Figure 2:**
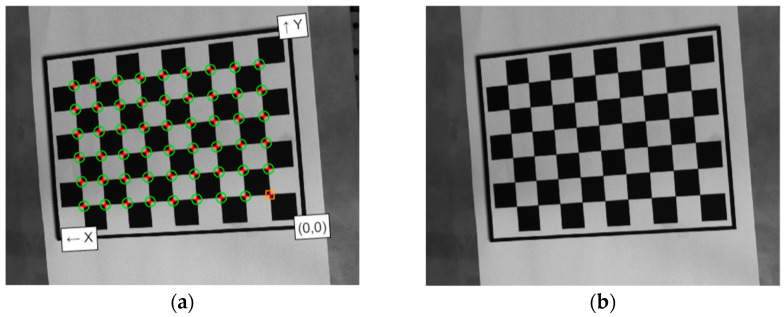
The recorded image: (**a**) before calibration; (**b**) after calibration.

The real-world coordinates of the measuring points on the object surface should be accurately mapped to the plane coordinates used in the camera settings during the vibration tests. This ensures that the absolute values (pixel displacement) obtained can be accurately converted to accelerations and compared to those measured using the accelerometer. To achieve this, a set of images of a planar calibration target with regularly spaced circular dots is captured using the imaging system at each position, with the target translated and/or rotated in three dimensions. These images are then processed to calculate the intrinsic and extrinsic parameters of the camera used. More details about the estimation of geometric parameters for a single camera can be found in various publications [22,23]. As shown in Figure 2, the recorded image is presented before and after calibration, which was performed using the MATLAB^®^ Camera Calibrator App (version 2022b). The measured pixel motion will subsequently be converted to actual displacement by applying the ratio between the observed pixels of the beam structure in the frame and the actual physical geometry parameters of the beam, after applying the obtained calibration parameter matrix.

### 3.3. Segmentation Map

**Figure 3:**
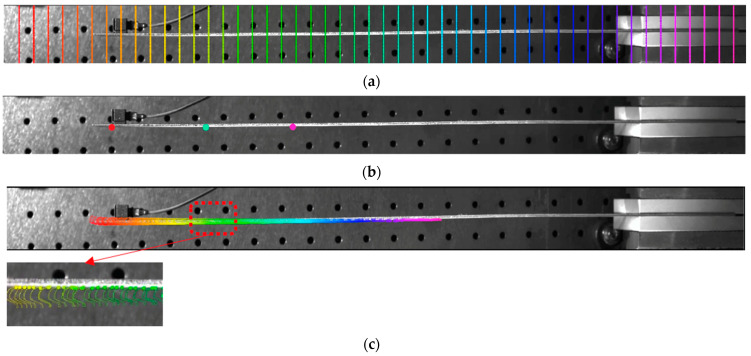
The three ways of designing tracking points (noted as rainbow dots): (**a**) regular grid-based tracking; (**b**) manually picked point-based tracking; (**c**) user-defined segmentation-based tracking (used in this paper): the trajectory plot of the edge points (including 15 frames) with a zoom-in view of the region marked by red dash box.

The CoTracker algorithm requires a grayscale contrast between the target and the background [18]. In images, the edges of objects contain the most information, as edges represent abrupt changes in grayscale values. Therefore, the segmentation map has been designed using the Canny edge detection method to cover the edges of the observed beam structure. Figure 3c presents the motion trajectory (including the first 15 frames) of all the points on the identified edge under a hammer impact, with a zoomed-in region. The extracted pixel motions of these points, in a total of 1207 pixels, are combined into a spatiotemporal matrix, which is later used for modal parameter extraction after applying the camera calibration parameters from Section 3.2. Figure 3a,b present the other two methods offered using CoTracker in tracking points: regular grid-based tracking and manually picked point-based tracking.

### 3.4. Modal Analysis Using Delay-Embedding Dynamic Mode Decomposition (DMD)

DMD is a data-driven decomposition technique [19]. It is a dimensionality reduction and modeling approach initially developed within the fluids community. Later, its inherent merit in the ability to decompose a matrix of high-dimensional time series data into a set of spatially coherent structures that exhibit the same linear dynamics over time (e.g., oscillations, exponential growth/decay) extended its application to fields such as video surveillance [20], epidemiology [21], and disease modeling [22]. However, when the spatial complexity is smaller than the spectral complexity, the standard DMD may not always yield the expected results, particularly in scenarios involving highly noisy data. A classic technique for dealing with long-term temporal behavior and high-dimensional noisy datasets is delay-embedding DMD. This method combines the ideas of classical DMD and a delay-embedding theorem [23] to augment a more versatile and robust state represented by a few measurement functions, with its former time-lagged snapshots for dynamic mode decomposition in various applications.

To implement delay-embedding DMD, stacking *S* ≤ *K* copies of time-shifted data is performed first to form the following augmented input matrix:

(1) $$\left.D_{aug}=\left(\begin{array}{cccc} x_{1} & x_{2} & \ldots & x_{K-S+1}\\ x_{2} & x_{3} & \ldots & x_{K-S+2}\\ \vdots & \vdots & \ddots & \vdots \\ x_{S} & x_{S+1} & \ldots & x_{K} \end{array}\right.\right)$$

where $x(t)\in R^{m}$ denotes a state vector of an *m*-dimensional dynamical system at time t. $x_{k}=x\left(t_{k}\right),\;$ for k=1,......K, a set of temporally equispaced snapshots with $t_{k}=t_{1}+(k-1)\Delta t$. *K* is the number of sample snapshots. And *S* is the applied embedding dimension.

A full snapshot matrix $X_{1}^{K}=\left[\begin{array}{cccc} x_{1} & x_{2} & \cdots & x_{K} \end{array}\right]$ is then constructed in the delay-embedding DMD. By superimposing standard DMD in a sliding window applied on the snapshot matrix$\;X_{1}^{K}$, with the sliding window moving from index 1 to *S*, snapshot subblocks $X_{1}^{K-S+1},X_{2}^{K-S+2},....,X_{S}^{K}$ are obtained from the full snapshot matrix $X_{1}^{K}$. $D_{aug}$ is arranged into the following two augmented matrices:

(2) $$X=\left[\begin{array}{c} X_{1}^{K-S+1}\\ X_{2}^{K-S+2}\\ \cdots \\ X_{S-1}^{K-1} \end{array}\right],\;\;\;\;X^{\prime }=\left[\begin{array}{c} X_{2}^{K-S+2}\\ \cdots \\ X_{S-1}^{K-1}\\ X_{S}^{K} \end{array}\right]$$

The goal of the delay-embedding DMD approach is to find a relationship between the future state $X^{\prime }$ and the current state X, given by the following:

(3) $$X^{\prime }=RX$$

where

(4) $$R=X^{\prime }X^{†}$$

X and $X^{\prime }$ are the augmented matrices, $X^{†}$ denotes the pseudo-inverse matrix of X. The matrix, R, is referred to as delay-embedding DMD operator. The delay-embedding DMD modes and eigenvalues are defined as the eigenvectors and eigenvalues of ***R***. A truncated singular value decomposition (SVD) is applied to X, then $X=U\Sigma V^{*}$, and ^*^ denotes Hermitian transpose of a matrix V. The pseudo-inverse matrix can be computed as follows:

(5) $$X^{†}=V\Sigma^{-1}U^{*}$$

Defining the projected matrix of ***R*** via least-square fitting as follows:

(6) $$\tilde{R}≜U^{*}RU=U^{*}X^{\prime }V\Sigma^{-1}U^{*}U=U^{*}X^{\prime }V\Sigma^{-1}$$

The delay-embedding DMD eigenvalues μ are then obtained by performing eigenvalue decomposition:

(7) $$\tilde{R}w=\mu w$$

And the delay-embedding DMD modes corresponding to μ is defined by the following:

(8) $$\phi =Uw=X^{\prime }V\Sigma^{-1}w$$

Denoting the *i*-th delay-embedding DMD eigenvalue as $\mu_{i}$, the growth rate $\delta_{i}$ and the frequencies $\omega_{i}\;$ are given by the following:

(9) $$\delta_{i}+i\omega_{i}=\frac{log\left(\mu_{i}\right)}{\Delta t}=s_{i}$$

Therefore, natural frequencies $f_{i}$, and modal damping ratios $\zeta_{i}$ can be extracted as follows:

(10) $$f_{i}=|s_{i}|/2\pi$$

(11) $$\zeta_{i}=-Re\left(s_{i}\right)/\left|s_{i}\right|$$

**Figure 4:**
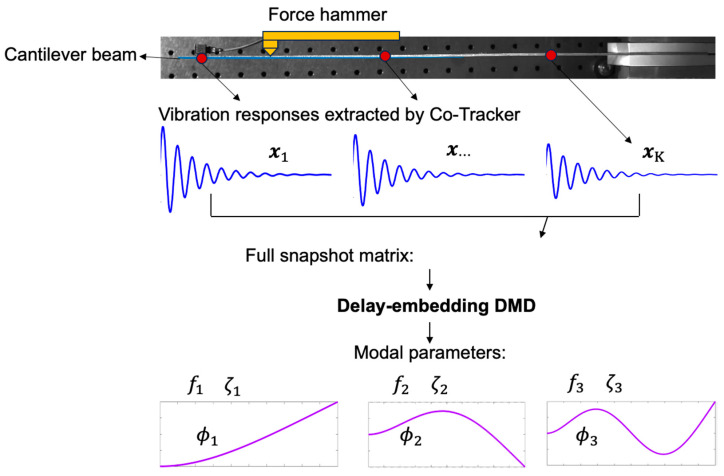
Schematic architecture of modal analysis using delay-embedding DMD.

The schematic architecture of modal analysis of the beam using delay-embedding DMD, based on the extracted vibration responses along the edge pixels covered by the segmentation map from Co-Tracker, is illustrated in Figure 4.

## 4. Results and Discussions

### 4.1. Modal Analysis Using Delay-Embedding Dynamic Mode Decomposition (DMD)

**Figure 5:**
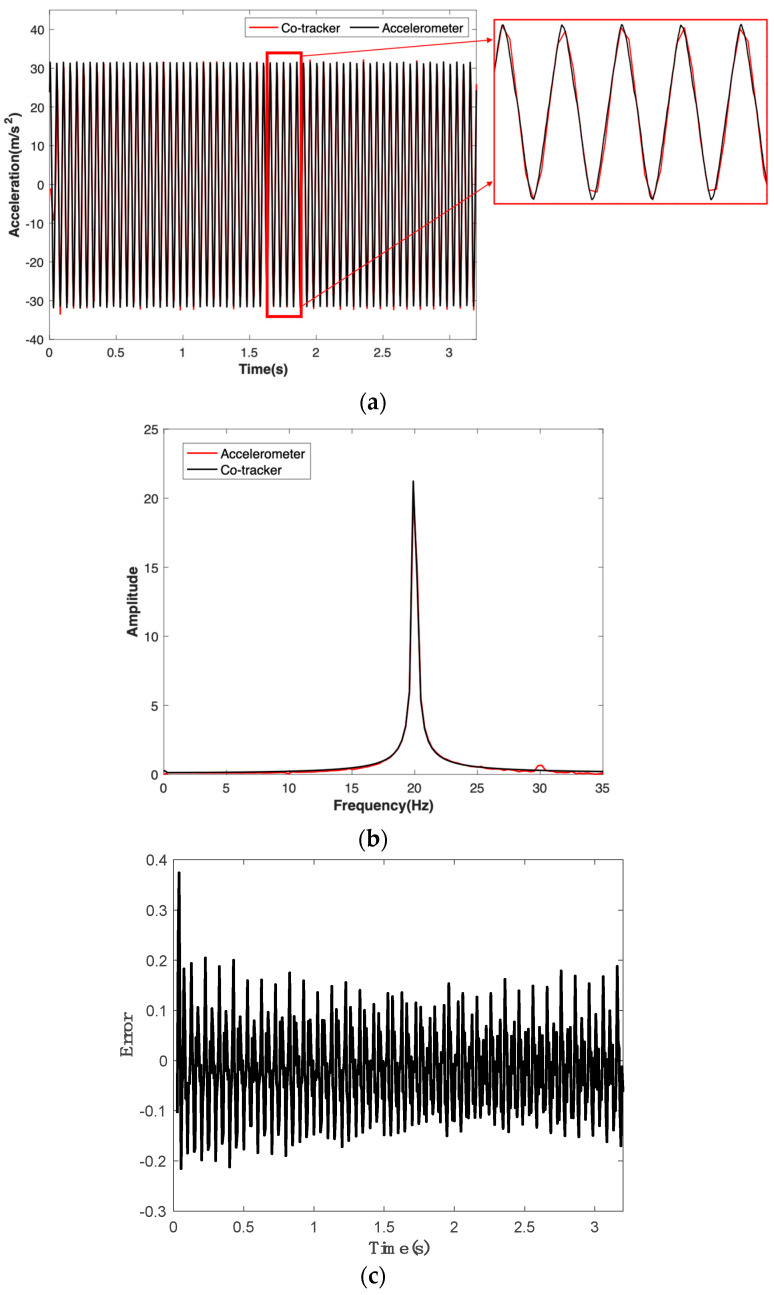
The comparison between vibrational acceleration responses from the CoTracker model and accelerometer results under a harmonic test at 20 Hz: (**a**) time−domain; (**b**) frequency−domain; (**c**) measurement differences between the CoTracker and the accelerometer.

To illustrate the feasibility and performance of using CoTracker to identify structural vibrations, a test was conducted on a cantilever beam (as shown in Figure 3) subjected to a harmonic excitation at 20 Hz using a modal shaker. The vibrational acceleration sensor data and CoTracker data, compared in the time-domain, are shown in Figure 5a. Note that the direct measurement from CoTracker is pixel displacement, and the corresponding acceleration is obtained via camera calibration, followed by applying the ratio of structural geometry in pixels to physical dimensions. The results indicate a good match between the two throughout the entire duration. For a quantitative comparison, the correlation reflecting the degree of association between the camera and accelerometer acceleration signals has been calculated to be over 90%. This further confirms the consistency between the acceleration signals extracted from the video using CoTracker and those measured using the accelerometer. Additionally, the frequency analysis of the acceleration data for both cases was conducted by performing a Fourier transform, as illustrated in Figure 5b. From Figure 5b, although there is a slight energy peak located around 30 Hz for CoTracker, it can be observed that CoTracker accurately identified the dominant frequency component around 20 Hz, which is identical to that of the accelerometer. The measurement difference with a mean value of −0.0225 and standard variation of 0.0970 between the Co-Tracker and the accelerometer is presented in Figure 5c.

### 4.2. Modal Parameters Identification under Hammer Impact

Since the comparison study in Section 4.1 validates the satisfactory performance of measuring structural vibrations using the AI model CoTracker, this section aims to explore the potential of identifying modal parameters, including natural frequency, damping ratio, and mode shape, based on the extracted vibrational signals from CoTracker. To achieve this, a hammer impact test is designed, as shown in Figure 4. The accelerations of 1207 pixels from the beam edge covered by the segmentation map are extracted using CoTracker. Subsequently, delay-embedding DMD is applied to these time signals to extract the aforementioned modal parameters.

**Figure 6:**
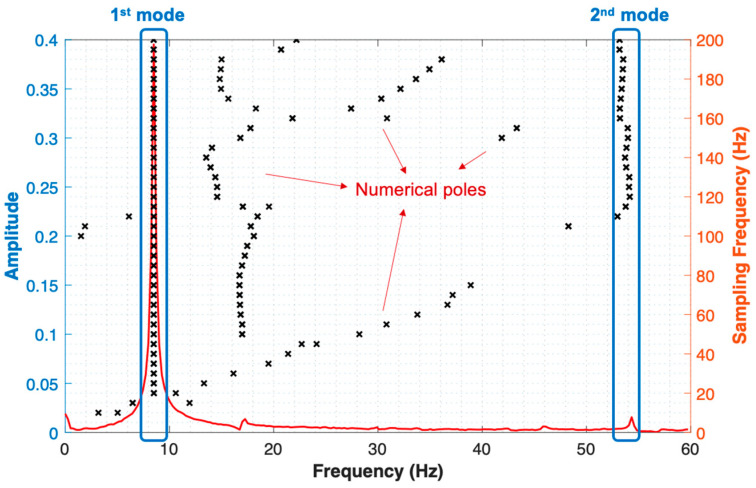
The pseudo-frequency stabilization diagram using delay-embedding DMD.

**Figure 7:**
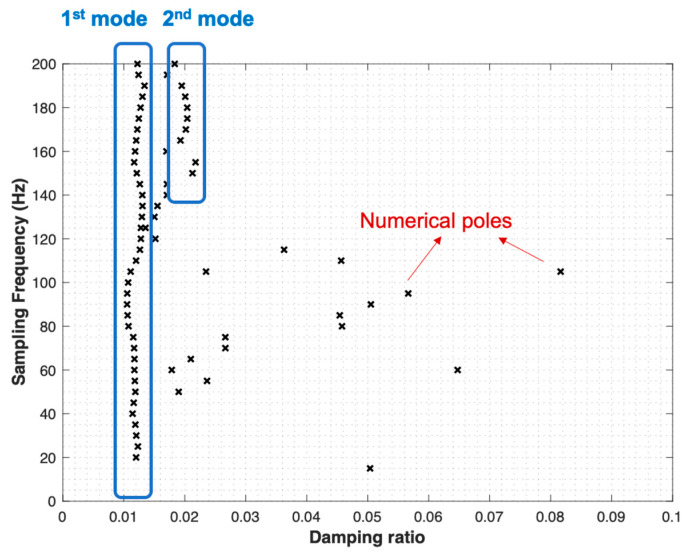
The pseudo-damping stabilization diagram using delay-embedding DMD.

The input data matrix (full snapshot matrix) is constructed with dimensions of (number of delays × number of pixels) × number of sampling points. In this case, the number of delays (*d*) is set to 10, the number of pixels is 1207, and the number of sampling points is 601, with a sampling frequency of 120 Hz. The modal parameters have then been extracted using the DMD procedure described in Section 3.4 To construct a similar stabilization diagram in LSCF [24], this paper employs the idea of a pseudo-frequency/damping stabilization diagram from recent research [25]. Instead of increasing the mode order in the stabilization diagram of the LSCF method, this paper considers the stabilization of DMD eigenvalues by increasing the sampling frequency using a resampling strategy. The sampling frequency in this paper ranges from 5 Hz to 200 Hz, increasing by 5 Hz increments. The pseudo-frequency and damping stabilization diagrams are shown in Figure 6 and Figure 7, respectively.

As can be seen from Figure 6, two stable modes at 8.5 Hz and 53 Hz, corresponding to stable damping ratios 0.012 and 0.020 in Figure 7, can be captured. Still, sparse numerical poles can be observed in both pseudo-frequency and damping stabilization diagrams, due to numerical calculations and noise. To validate the correctness of the two identified modes, the numerical solution of the cantilever beam of the first two natural frequencies and the mode shapes of flexural vibration is calculated in the commercial Finite Element Method (FEM) software COMSOL^®^ Version 6.2.

The FEM results indicate that the first two natural frequencies are 9 Hz and 56 Hz, respectively, considering the Young’s modulus and density of the beam as 190 GPa and 7850 $kg/m^{3}$, which demonstrates a good match with the identified DMD frequencies. Furthermore, the DMD mode shapes, after a procedure of outlier removing and smooth averaging of the identified two modes, are presented in Table 1, which again shows a consistency with the corresponding FEM mode shapes.

## 5. Conclusions

This paper first explores the feasibility of using the current state-of-the-art tracking deep learning model, CoTracker, for measuring the structural vibrations of a cantilever beam structure. The measured results are compared with the acceleration data from a reference accelerometer. The comparison indicates the promising functionality of CoTracker in vibration measurement, particularly in terms of time/frequency measurement accuracy. Furthermore, a data-driven approach called delay-embedding DMD has been employed to identify the underlying flexural vibration modes, including natural frequencies and damping ratios. By leveraging the full-field capability of camera-based measurements at the pixel-level, the mode shapes can also be recognized using CoTracker. The identified modal parameters show satisfactory consistency with FEM solutions. This preliminary study confirms the effectiveness of integrating deep learning technologies into engineering problems in structural vibration domains. This illustrates its high potential in broader dynamics fields such as vibration control based on camera, motion magnification, and nonlinear dynamics. In the near future, camera-based measurement experiments on more complex and practical engineering structures will be conducted to further investigate the actual applicability of CoTracker and DMD in acquiring vibrations and identifying structural modal parameters.

## Figures and Tables

**Table 1:** The 1st and 2nd mode shapes based on DMD and FEM.

1st DMD mode shape	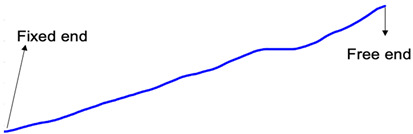
1st mode shape from COMSOL	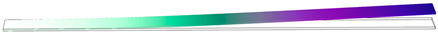
2nd DMD mode shape	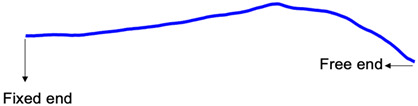
2nd mode shape from COMSOL	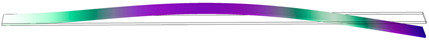

## Data Availability

The raw data supporting the conclusions of this article will be made available by the authors on request.
